# Screening of molecular elements and improvement of heat resistance in a thermophilic bacterium

**DOI:** 10.1016/j.engmic.2025.100225

**Published:** 2025-07-22

**Authors:** Jie Cui, Caifeng Li, Gongze Cao, Yuxia Wu, Shouying Xu, Youming Zhang, Xiaoying Bian, Qiang Tu, Wentao Zheng

**Affiliations:** a State Key Laboratory of Microbial Technology, Shandong University, No. 72 Binhai Road, Qingdao 266237, China; b Microbial Oil Recovery Research Institute, Research Institute of Petroleum Engineering, Shengli Oilfield Company, Sinopec, Dongying 257000, China; c Institute of Synthetic Biology Industry, Hunan University of Arts and Science, Changde 415000, China; d Shenzhen Research Institute of Shandong University, A301 Virtual University Park in South District of Shenzhen, Guangdong 518000, China

**Keywords:** Geobacillus stearothermophilus, Bacterial thermotolerance, Adaptive laboratory evolution, Engineering microorganism

## Abstract

Engineering microorganisms to withstand extreme temperatures (>80 °C) remains a critical challenge in industrial biotechnology owing to limited genetic tools and poor mechanistic understanding of microbial thermoadaptation. We aimed to develop a novel *Geobacillus stearothermophilus* strain with remarkable thermal resilience through an integrated approach combining adaptive laboratory evolution and rational genetic engineering. Progressive thermal adaptation (70–80 °C) followed by genome reduction generated a mutant (SL-1–80) with enhanced stability at 80 °C. Subsequent combinatorial overexpression of eight heat-associated genes (*murD, cysM, grpE, groES, hsp33, hslO, hrcA, clpE*) synergistically extended its survival to 85 °C. Genomic and transcriptomic analyses revealed a triple mechanism: (1) strategic deletion of transposable elements (IS5377/IS4/IS110) reduced genomic instability, (2) co-activation of chaperone systems (GroES-GrpE) and redox homeostasis enzymes (HslO—Hsp33) enhanced protein folding and oxidative stress resistance, and (3) metabolic plasticity (BglG and HTH-domain transcriptional repressor), motility optimization (FliY), and transcriptional reprogramming (Sigma-D, DUF47-family chaperone and HTH-domain transcriptional repressor) facilitated nutrient acquisition and motility-based environmental navigation under stress. Furthermore, we established the first high-efficiency electroporation protocol (10^4^ transformants/µg DNA) for this genus, enabling ATP-enhanced heterologous protein expression under heat stress. This study provided a robust platform organism for high-temperature bioprocessing and a mechanistic blueprint for engineering microbial thermotolerance, addressing key limitations in applications such as microbial-enhanced oil recovery and industrial enzyme production.

## Highlights

• Engineered *G. stearothermophilus* SL-1-H1 survives at 85 °C via gene overexpression.
• Developed first efficient electroporation for *Geobacillus* (10⁴ CFU/µg DNA).
• Synergistic gene overexpression (e.g., GroES-GrpE) boosted thermotolerance.
• ALE & genome reduction created base mutant (SL-1–80) stable at 80 °C.
• Genomic changes promoted motility, metabolism & stress response under heat.

## Graphical abstract

**Figure:**
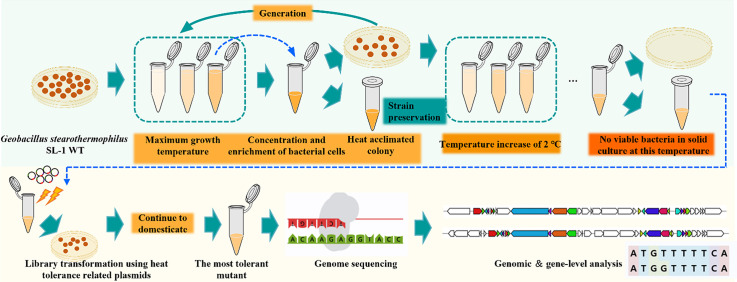

## 1 Introduction

Global energy demand continues to grow at an unprecedented pace, driven by population growth, improved living standards, and industrialization of emerging economies [1]. Despite significant investment in renewable energy systems, such as biofuels, solar, and wind energy, fossil fuels remain the dominant energy source worldwide [2]. The reliance on fossil fuels is further strained by the rapid depletion of conventional light crude oil reserves, which fails to meet the requirements of global consumption. Inefficient extraction technologies further exacerbate the widening supply-demand gap. Therefore, unconventional hydrocarbon reserves, such as heavy oil, asphalt, and oil sands, have emerged as critical alternatives for fossil fuel resources. However, their exploitation remains economically and technically challenging owing to intrinsic physicochemical limitations, such as their high viscosity, density, and capillary forces, which hinder the application of conventional extraction methods [3,4]. Microbial enhanced oil recovery (MEOR) which leverages biological processes to improve oil mobility and recovery efficiency, has thus emerged as a cost-effective technology for oil extraction from unconventional hydrocarbon reserves [5].

In MEOR technology, microorganisms enhance oil recovery by altering reservoir rock and crude oil properties through their metabolic activities and migration, effectively mobilizing trapped oil in depleted or high water-cut reservoirs. Compared to conventional methods, this approach demonstrates notable cost-effectiveness, environmental compatibility, and operational adaptability [[6], [7], [8]]. Current MEOR implementations primarily adopt two strategies: (1) direct injection of microbial fermentation products as biochemical enhancers, and (2) stimulation of indigenous microorganisms by injecting nutrient solutions to promote metabolic activity, where microbial cells or their byproducts interact with the oil layer to improve recovery rates [9]. Essential prerequisites for successful MEOR applications include microbial viability under reservoir conditions (e.g., high temperature and pressure), bioproduction capacity of functional metabolites (e.g., viscosity reducers, emulsifiers), and the effectiveness of biological processes in displacing residual oil [10,11]. Despite the recent progress, the broader development of MEOR has substantial challenges, particularly in obtaining or engineering microbial strains that combine environmental resilience and stability with high metabolic activity under extreme reservoir conditions [12].

*Geobacillus stearothermophilus*, a thermophilic gram-positive bacterium of the phylum Firmicutes, exhibits unique adaptability to extreme environments. This genus is renowned for its heat tolerance, solvent resistance (e.g. ethanol) [[13], [14], [15], [16]], hydrocarbon degradation/utilization capabilities [[17], [18], [19]], and heavy metal ion tolerance [20], rendering it valuable for enzyme engineering, biodegradation, and bioremediation. Notably, as indigenous inhabitants of oil reservoirs, *Geobacillus* species play critical roles in native microbial consortia. For example, *G. thermoglucosidasius* SL-1, isolated from the Shengli Oilfield, demonstrates crude oil displacement efficacy [21]. Strengthening the thermal resilience of *G. stearothermophilus* SL-1 can increase microbial survival and proliferation under reservoir conditions, thereby improving oil recovery efficiency and expanding MEOR applicability.

Adaptive laboratory evolution (ALE), which accelerates natural evolutionary processes in vitro to obtain targeted phenotypes, has been successfully used to improve thermotolerance in diverse bacteria [[22], [23], [24], [25], [26]]. While genetic modification offers a direct route for engineering the desired traits, molecular tools for genetic modification of *G. stearothermophilus* are currently underdeveloped. This study addresses the gap by characterizing functional genetic elements in *G. stearothermophilus* and synergistically integrating ALE with genetic engineering. Through thermal adaptation experiments, we successfully acclimatized a mutant *G. stearothermophilus* strain for growth at 85 °C. Subsequent genome sequencing identified critical genes linked to heat resistance mechanisms. These findings provide a foundational framework for thermal adaptation engineering of industrially essential thermophilic bacteria.

## 2 Materials and methods

### 2.1 Strain, culture medium and antibiotics

The *G. thermoglucosidasius* SL-1 strain used in this study was isolated from a water-flooded well in the Shengli Oilfield of Sinopec (37.46°N, 118.48°E), Dongying, Shandong Province, China. Two types of media were primarily used in this study: low-salt Luria-Bertani (LB) medium, 2 × LBG medium. Additional media details are summarized in **Table S1**. The following reagents were used: inducer-l-arabinose (Ara) and antibiotics, including apramycin (Apra), chloramphenicol (Cm), gentamicin (Genta) and kanamycin (Km) (complete specifications are provided in **Table S2**). All chemical reagents were obtained from Sinopharm Chemical Reagent Co., Ltd (Shanghai, China). and all antibiotics were sourced from Shanghai Sangong Bioengineering Co., Ltd (Shanghai, China).

### 2.2 Strain culture and plasmid construction

Plasmid construction in this study utilized *Escherichia coli* Red/ET recombination engineering. The plasmids were constructed in *E. coli* GB05-dir, a derivative strain based on *E. coli* HS996 with the knockout of chromosomal *recET* and *ybcC* genes and insertion of *recE, recT, redγ* and *recA* genes under P_BAD_ promoter control at the *ybcC* locus. GB05-dir was inoculated in antibiotic-free LB medium at 37 °C with shaking at 950 rpm. For comparison and plasmid propagation, *E. coli* GB05 (the parental strain without genomic insertions) served as a chassis for exogenous plasmids. GB05 was cultured under identical conditions to those used for GB05-dir. For thermophilic bacterial culture, wild-type *G. stearothermophilus* SL-1 was maintained in antibiotic-free LB medium at 65 °C with shaking at 950 rpm. Plasmid-bearing strains were cultured in LB medium supplemented with appropriate antibiotics.

For the preparation of competent GB05-dir cells, an overnight culture was diluted (1:300) in fresh LB medium and cultured for 2 h at 37 °C with shaking at 950 rpm. Following induction with Ara (35 µL) for 40 min, the cells were washed twice with sterile water and finally resuspended in residual supernatant (30 µL). DNA fragments containing 50 bp homology arms at both 5′ and 3′ ends, which facilitated in vivo recombination with adjacent fragments, were introduced into GB05-dir competent cells via electroporation. Electroporation was performed in 1 mm cuvettes at 1350 V; subsequently, antibiotic-free LB medium (1 mL) was immediately added for cell recovery, followed by incubation at 37 °C with shaking at 950 rpm for 1 h. The transformed cells were then plated on LB agar containing appropriate antibiotics for selection. Recombinant colonies were subsequently isolated for plasmid extraction, and successful plasmid construction was verified through restriction enzyme digestion and next-generation sequencing analysis.

### 2.3 Thermostable antibiotic marker selection in G. stearothermophilus SL-1

Based on the characteristics of *G. stearothermophilus* SL-1, we conducted antibiotic sensitivity testing using a panel of antibiotics including streptomycin (commonly effective against *Bacillus* species), erythromycin and tetracycline (targeting resistant strains), along with Apra, Kan, Genta, and Cm (commonly used in laboratory). Antibiotic resistance was evaluated using LB agar plates containing concentration gradients of each antibiotic (1, 5, 10, 15, 20, 30, 50, 80, and 100 µg·mL⁻¹). For testing, 50 µL aliquots of an overnight *G. stearothermophilus* SL-1 culture were spread onto triplicate plates for each antibiotic concentration. Following 48-h incubation at 65 °C, colony growth was examined to determine resistance profiles.

### 2.4 Determination of electroporation conditions in G. stearothermophilus SL-1

Different replicon plasmids carrying sensitive antibiotic resistance genes *of G. stearothermophilus SL*-1 were used as substrates. Electroporation was performed according to the conditions listed in **Table S3,** and successful transformants were identified on selective antibiotic plates. To optimize the transformation efficiency, we systematically evaluated the electroporation process under multiple experimental conditions, including transformation temperature, buffer composition, applied voltage, electroporation cuvette size, recovery medium formulation, as well as post-transformation incubation temperature and duration.

### 2.5 Screening of heat-resistant fluorescent reporter genes in G. stearothermophilus SL-1

The codon-optimized fluorescent reporter genes for GFP (green fluorescent protein) and YFP (yellow fluorescent protein) were integrated into working plasmids of *G. stearothermophilus* SL-1, with their expression driven by a functional resistance gene promoter native to this strain. After transforming these reporter plasmids into *G. stearothermophilus* SL-1, we cultured the transformants in selective medium until mid-log phase. Fluorescence detection was performed using laser confocal microscopy, with GFP and YFP transformants analyzed at 488 nm excitation/530 nm emission and 513 nm excitation/531 nm emission wavelengths, respectively. These experiments were conducted to determine the functionality of these reporter systems in this thermophilic strain.

### 2.6 Characterization of endogenous promoters in G. stearothermophilus SL-1

Then, the transcriptome of *G. stearothermophilus* SL-1 across three growth phases (mid-logarithmic, stationary, and late logarithmic) was analyzed to identify potential endogenous promoters. From the top 300 most highly transcribed genes in each phase (ranked by FPKM values), the 50 candidate genes meeting specific criteria were selected and the intergenic sequences between their start codons and the upstream genes' stop codons were extracted. These sequences, designated as SP1-SP50 promoters, were constructed into working plasmids in *E. coli* to control the expression of fluorescent reporter genes. After transforming these constructs into *G. stearothermophilus* SL-1, the intensity of the promoters was determined using flow cytometry and laser confocal microscopy. For laser confocal microscopy screening, kanamycin-resistant transformants were cultured aerobically in LB medium at 65 °C overnight, harvested using centrifugation (3000 rpm, 5 min), and resuspended in fresh medium (200 µL). To maintain cell viability during imaging, samples were incubated at 50-70 °C in a metal bath before preparing microscope slides with 10 µL aliquots of bacterial suspension. This systematic approach enabled identification of promoters with varying expression intensities, providing a valuable toolkit for genetic engineering and optimized gene expression in *G. stearothermophilus* SL-1.

### 2.7 Determination of the wild- type growth temperature range in G. stearothermophilus SL-1

To characterize the thermal growth profile of wild-type *G. stearothermophilus* SL-1, a single colony was inoculated into LB medium (1.8 mL) and pre-cultured at 60 °C with shaking at 960 rpm for 12 h. A 30 µL aliquot of this pre-culture was then transferred to fresh LB medium (1.8 mL) and incubated across a temperature gradient series (40 °C, 45 °C, 50 °C, 55 °C, 60 °C, 65 °C, 70 °C, and 75 °C) with consistent shaking at 960 rpm for 12 h at each temperature. Growth was quantified by measuring the optical density at 600 nm (OD_600_) for all temperature conditions. This systematic temperature profiling enabled precise determination of the strain's optimal growth range and thermal tolerance limits.

### 2.8 Acquisition of heat-resistant mutants by adaptive laboratory evolution in G. stearothermophilus SL-1

ALE experiments were conducted on *G. stearothermophilus* SL-1 to develop heat-resistant mutants through sequential thermal acclimation and selection. The experimental protocol consisted of four key stages. First, wild-type cultures were incubated in LB medium (1.8 mL) at their optimal temperature for 16 h and plated on LB agar to prepare starter cultures. Second, individual colonies were inoculated into fresh LB medium (1.8 mL) and cultured at progressively increasing temperatures (starting from the maximum growth temperature) for 18 h with shaking at 960 rpm; OD_600_ measurements were used to identify the best-performing mutant in each cycle. Third, the remaining culture was concentrated using centrifugation, with one portion preserved in glycerol stocks while another portion was plated on LB agar at the test temperature. Fourth, the selection temperature was incrementally increased by 2 °C per cycle until the thermal limit for colony formation was attained. The surviving isolate from the highest achievable temperature was designated as the most thermotolerant mutant. This iterative ALE strategy successfully generated *G. stearothermophilus* SL-1 variants with enhanced heat resistance, demonstrating the effectiveness of this method for engineering microbial strains suited to extreme thermal environments.

The agar plates were prone to thinning owing to moisture evaporation during high-temperature incubation; hence, the following measures were implemented to address this issue: 1. Increased medium volume. During plate preparation, we used approximately 30 mL of LB agar medium per plate, which is greater than the standard amount used for mesophilic bacterial cultures. 2. Enhanced sealing. Plates were sealed with multiple layers of Parafilm. Parafilm can degrade and break owing to prolonged heat exposure; hence, we monitored and replenished the seals as needed. In some cases, the plates were also placed inside heat-resistant plastic bags to further minimize moisture loss. 3. Optimized incubation conditions. The plates were positioned in the incubator so as to avoid direct contact with metal surfaces, thereby preventing localized heat accumulation. Moreover, the plates were placed appropriately ensuring uniform heat distribution for consistent growth conditions. These precautions effectively maintained plate integrity throughout the extended incubation period.

### 2.9 Overexpression of heat tolerance-related genes improves heat tolerance of mutant strain in G. stearothermophilus SL-1

To systematically identify and enhance thermotolerance in *G. stearothermophilus* SL-1, a comprehensive approach involving genomic analysis, plasmid library construction, and ALE was employed. The experimental workflow comprised two primary phases. First, we conducted genome-wide identification and comparative analysis of known thermotolerance-related genes in *G. stearothermophilus* SL-1. These candidate genes were then cloned into an expression vector using Red/ET recombination technology, with each gene placed under the control of a strong constitutive promoter to maximize the expression levels in the host strain. Second, we utilized the most thermotolerant ALE-derived mutant as the transformation host, introducing the pooled plasmid library via electroporation to ensure comprehensive coverage of all genetic variants. Following recovery, the transformed cells were subjected to an 85 °C heat shock for 4 h before being plated on selective antibiotic plates and cultured at the starting strain's maximum growth temperature to identify heat-resistant transformants. Primary screening involved identifying colonies demonstrating vigorous growth as indicated by the OD_600_ measurements after culturing individual isolates in selective LB medium at elevated temperatures for 12 h. For promising candidates, 100 µL aliquots were transferred to fresh selective medium, with the incubation temperature progressively increased by 2 °C increments in the subsequent screening rounds until reaching the thermal limit for cell viability. The most thermotolerant mutant was selected for downstream analysis, with its plasmids extracted and transformed into *E. coli* GB05 for amplification. Plasmid content was analyzed by KpnΙ restriction digestion (recognition sites intentionally preserved in the vector backbone), enabling determination of plasmid copy number (**Figure S2**). Subsequently, plasmid-borne genes were identified through next-generation sequencing (NGS). This integrated approach combines genomic analysis with adaptive laboratory evolution (ALE) to systematically identify and enhance thermotolerance in *G. stearothermophilus* SL-1, establishing a robust framework for engineering industrial strains with superior heat resistance.

### 2.10 Comparative genomic analysis of heat-resistant mutants and wild type in G. stearothermophilus SL-1

Whole-genome sequencing was performed on both wild-type *G. stearothermophilus* SL-1 and its most thermotolerant mutant to identify genetic modifications conferring enhanced heat resistance. For genome annotation, coding sequences were predicted using GeneMarkS, a self-training algorithm for prokaryotic genomes that integrates GeneMark.hmm program with an iterative training procedure to optimize prediction parameters. Tandem repeats were identified using RepeatMasker, while genomic islands (potential hotspots for horizontally acquired adaptive genes) were detected with IslandPath-DIOMB. Secondary metabolite biosynthetic gene clusters were characterized using AntiSMASH, providing comprehensive insights into the strain's metabolic potential. SignalP predicted signal peptides critical for protein secretion, while TMHMM was used to identify transmembrane domains in encoded proteins. For virulence-related adaptations, EffectiveT3 was used to predict type III secretion system effectors known to mediate both pathogenicity and environmental stress responses. Comparative genomic analysis using Geneious Prime enabled comprehensive evaluation of genome architecture (size and gene content) and detailed sequence comparison between wild-type and thermotolerant mutant strains. The software's integrated tools for sequence alignment, annotation, and variant analysis facilitated precise identification of thermotolerance-associated mutations. This multi-dimensional bioinformatics approach elucidated key genetic determinants of heat resistance, establishing a molecular framework for targeted strain optimization and industrial bioprocess applications.

## 3 Results

### 3.1 Growth temperature range characterization and thermostable antibiotic marker selection in G. stearothermophilus SL-1

**Fig. 1:**
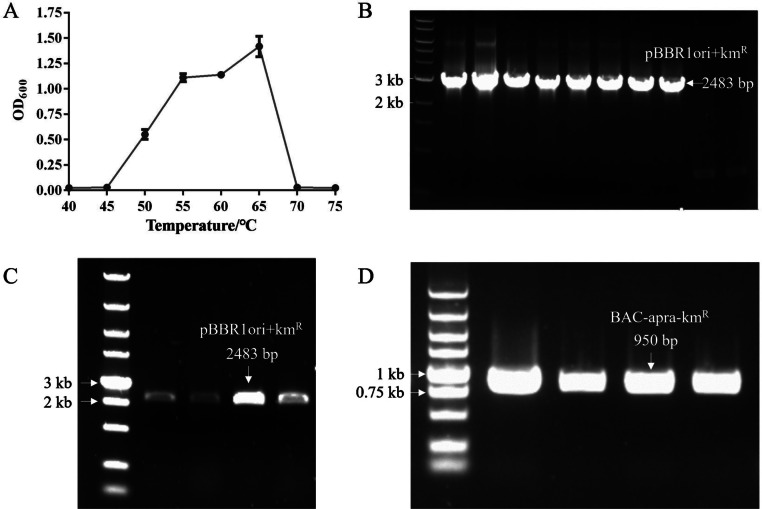
Thermotolerance and plasmid characterization of *G. stearothermophilus* SL-1. (A) Growth profile of wild-type SL-1 strain across its thermal tolerance range. (B) Verification of pBBR1-Km transformants using PCR amplification of the 2483 bp fragment (pBBR1 origin + kanamycin resistance cassette, pBBR1 ori + km^R^). (C) Stability assessment of pBBR1-Km through subculturing, confirmed using 860 bp kmR gene amplification. (D) Validation of BAC-Apra-Km transformants via PCR detection of the 950 bp resistance cassette.

A single-factor experiment assessed the impact of culture temperature on the growth of wild-type *G. stearothermophilus* SL-1. The results revealed a temperature-dependent growth pattern: biomass accumulation initially increased with elevated temperatures, peaked at 65 °C, and subsequently declined. This established 65 °C as the optimal growth temperature. The strain exhibited a well-defined thermal tolerance range, with active proliferation observed between 45 °C and 70 °C, whereas temperatures 〈 45 °C or 〉 70 °C completely inhibited growth (Fig. 1A).

*G. stearothermophilus*, a Gram-positive obligate thermophilic within the phylum Firmicutes, requires antibiotic selection markers for genetic manipulation under high-temperature conditions. Given the limited availability of thermostable antibiotics suitable for screening, we first systematically screened antibiotic resistance profiles during the development of its DNA transformation system to identify viable selection markers. The results indicated that *G. stearothermophilus* SL-1 was highly sensitive to most tested antibiotics, with aminoglycosides and macrolides (e.g., erythromycin) exhibiting pronounced growth inhibition (**Table S2**).

### 3.2 Development of a genetic transformation system for G. stearothermophilus SL-1

The development of a genetic transformation system for *Geobacillus* species faces significant challenges owing to their unique physiological and structural characteristics. The three major barriers are as follows: (1) thick peptidoglycan layer: as Gram-positive bacteria, *Geobacillus* species are encased in a dense peptidoglycan cell wall that physically impedes exogenous DNA entry; (2) low permeability of plasma membrane: the inherently low permeability of their plasma membrane further restricts extracellular DNA uptake; (3) thermophilic adaptation: their high-temperature growth environment (45–70 °C) compromises the stability and functionality of genetic tools derived from mesophilic microorganisms. These interrelated constraints collectively hinder the establishment of effective genetic manipulation platforms across the *Geobacillus* genus.

Among current *Bacillus* transformation methods [13,14,16,27,28], electroporation is remarkable for its operational simplicity, eliminating requirements for paired donor bacteria or extensive buffer/culture medium pretreatments. To establish an electroporation system for *G. stearothermophilus* SL-1, we first tested the *Bacillus*-adapted replicon plasmid pPJK-Cat under modified Gram-negative electroporation protocols. Key parameters such as buffer composition, electric pulse conditions, recovery medium, recovery temperature, and recovery time were systematically optimized **(Table S3)**. However, no pPJK-Cat transformants were obtained. Subsequently, we expanded the selection of replicons using four plasmids from our laboratory repository (BAC-Apra-Km, RK_2_-Apra-Cm, P15A-Cm, and pBBR1-Rha-Redγ-Redαβ-Km). Following iterative parameter adjustments, successful transformations were achieved with the BAC-Apra-Km and pBBR1-Rha-Redγ-Redαβ-Km, and validated using colony PCR (Fig. 1B and D). To assess plasmid stability, transformants underwent 10 generations of subculturing at 65 °C. Persistent plasmid detection post-culturing confirmed stable replication (Fig. 1C).

These findings demonstrate that BAC-Km and pBBR1-Km plasmids maintain functionality and stability during replication in *G. stearothermophilus* SL-1. Their robustness makes them reliable genetic tools for engineering this thermophilic bacterium. The establishment of this electroporation-based transformation system addresses a critical constraint in *Geobacillus* genetic manipulation, providing a foundational platform for biotechnological applications and advancing fundamental studies. The finalized protocol for *G. stearothermophilus* SL-1 electroporation comprises the following steps: (1) Initial culture preparation: A single colony is selected from a plate that has been incubated for 24 h. Then, the colony is inoculated into LB medium (1.8 mL). The inoculated medium is cultured at 65 °C with shaking at 960 rpm for 12 h. (2) Subculture preparation: The overnight culture solution (50 µL) was inoculated into fresh medium (1.8 mL). It was cultured at 65 °C with shaking at 960 rpm until the OD_600_ reaches 1.0. (3) Bacterial collection and resuspension: The bacteria is selected using centrifugation at 6000 rpm for 1 min at 4 °C. Then, the bacterial pellet is resuspended with pre-cooled water (1 mL) as a buffer. Subsequently, the centrifugation is repeated and resuspended three times. Finally, the bacteria are resuspended in water (50 µL). (4) DNA addition and electroporation: Desalted DNA (300–1000 ng) is added to the resuspended bacterial solution and mixed well. The mixture is transferred to a sterile, dry 2 mm electroporation cuvette. The cuvette is placed on ice for 2 min. The cells are electroporated at 2000 V (10 kV/cm), 200 Ω, and 25 µF. (5) Recovery and incubation: Antibiotic-free 2 × LBG medium (1 mL) is added to the electroporation cuvette and mixed with the bacterial solution. The mixture transferred to a new 2 mL centrifuge tube. The culture is revived at 48 °C with shaking at 960 rpm for 2 h. After recovery, the cells are centrifuged at 6000 rpm for 1 min. The supernatant is discarded and approximately 100 µL of the medium is retained to resuspend the cells. The resuspended cells are inoculated on an LB plate containing the corresponding antibiotics. The plate is incubated upside down in a 65 °C incubator for 12 h to visualize colonies. This established protocol provides a systematic, temperature-adapted electroporation framework for *G. stearothermophilus* SL-1, ensuring efficient transformation and recovery of transformants under high-temperature conditions.

### 3.3 Thermostable endogenous promoter screening in G. stearothermophilus SL-1

**Fig. 2:**
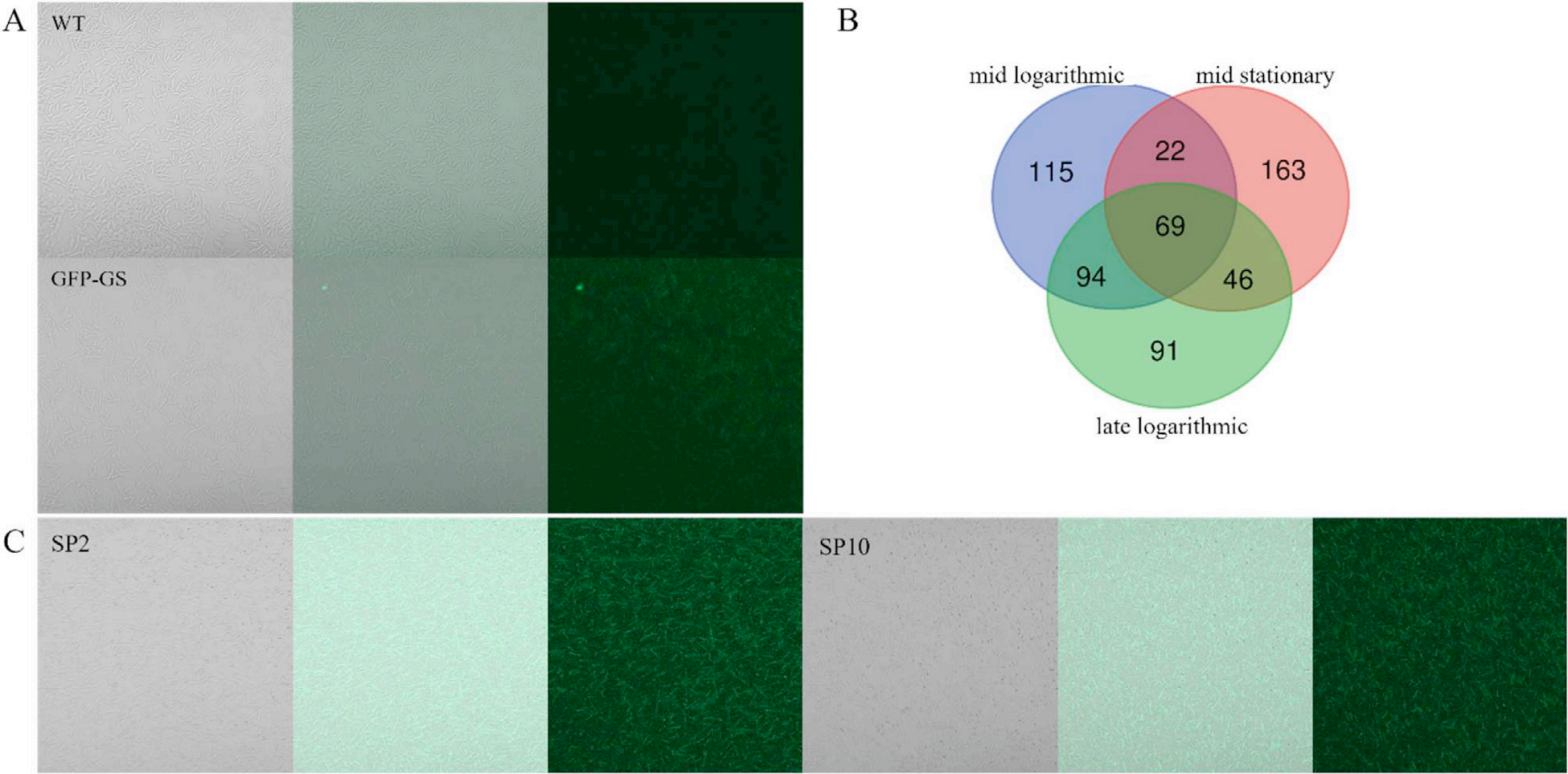
Endogenous promoter screening in *G. stearothermophilus* SL-1. (A) Confocal microscopy analysis of GFP-expressing transformants confirmed successful reporter system construction. (B) Transcriptomic profiling identified the top 300 highly expressed genes across three distinct growth phases. (C) Quantitative assessment of promoter strength was performed via confocal microscopy, demonstrating relative activity of SP2 versus SP10 constructs here (left to right respectively).

To screen functional promoters in *G. stearothermophilus* SL-1, we cloned codon-optimized fluorescent reporter genes (GFP-GS and YFP-GS) into the BAC-Km plasmid under the control of the kanamycin resistance gene promoter (P_km_). Following plasmid transformation into *G. stearothermophilus* SL-1, laser confocal microscopy revealed GFP-GS-derived fluorescence in bacterial cultures (Fig. 2A), confirming its utility as a functional reporter. By contrast, YFP-GS exhibited no detectable signal. This process confirmed that GFP-GS enables quantitative assessment of the promoter activity by correlating the fluorescence intensity to transcriptional strength when fused with candidate promoter sequences.

RNA-seq-derived FPKM analysis identified 50 highly transcribed genes across the mid-logarithmic, stationary, and late logarithmic growth phases of *G. stearothermophilus* SL-1 (Fig. 2B), ranking them based on the sum of their FPKM values. Promoter regions for library construction were defined as intergenic sequences spanning from the stop codon of the upstream open reading frame (ORF) to the start codon of each target gene, designated SP1-SP50. These candidate promoters, presented in **Table S4**, establish a genome mining-derived framework for endogenous promoter discovery. This strategy enables systematic identification of transcriptional regulatory elements in *G. stearothermophilus* SL-1, facilitating tunability for precise engineering applications in this thermophilic bacterium. Each candidate promoter (SP1-SP50) was cloned upstream of the GFP-GS fluorescent reporter in the BAC-Km-GFP-GS-terminator vector to drive expression. Transformants exhibiting visible fluorescence were selected for further analysis (Fig. 2C).

Initial trials using overnight cultures revealed extensive sporulation or cell death in *G. stearothermophilus* SL-1 populations. To mitigate this issue, growth durations of 6, 8, 10 and 12 h were systematically evaluated. Laser confocal microscopy detected GFP fluorescence in 8-, 10-, and 12-h cultures (Fig. 2C), with fluorescence-positive transformants categorized according to the incubation period in **Table S5.** This establishes an 8–12 h post-inoculation timeframe as optimal for promoter activity assessment via the GFP-GS reporter system, obtaining cells for reliable functional promoter identification.

### 3.4 Enhancing fluorescent protein expression through ATP supplementation and quantifying promoter strength

The observed weak fluorescence intensity and limited number of fluorescent transformants suggested compromised bacterial viability under high-temperature incubation. To mitigate energy depletion, we supplemented the culture medium with ATP at graded concentrations (0-1.2 mM). Transformants harboring different promoters were cultured aerobically at 65 °C for 6–12 h in ATP-amended media, followed by fluorescence quantification via laser confocal microscopy. Cultivation with 1 mM ATP for 12 h yielded the most significant enhancement of fluorescence protein expression. Under the optimized culture conditions, fifteen promoters (SP2, SP4, SP6, SP10, SP19, SP20, SP30, SP33, SP34, SP36, SP42, SP43, SP45, SP47, and SP49) successfully drove detectable GFP-GS fluorescence. Fluorescence-positive transformant counts across experimental conditions are cataloged in **Table S6**.

**Fig. 3:**
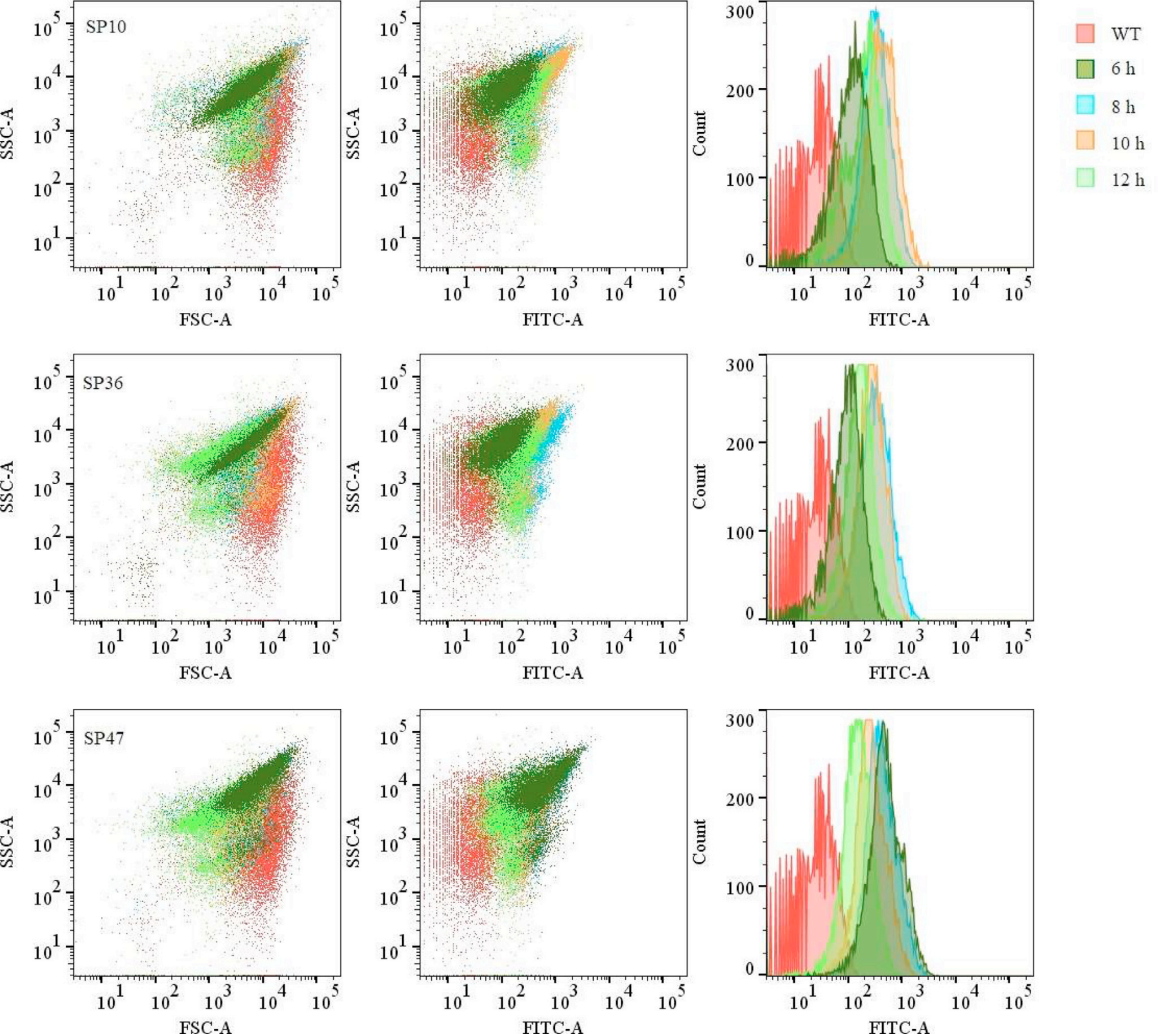
Quantification of promoter activity in *G. stearothermophilus* SL-1 WT under 1 mM ATP induction at distinct growth phases was performed via flow cytometric analysis. Among 18 screened promoters, SP10, SP36, and SP47 exhibited sustained high transcriptional activity across multiple growth stages and are shown here. The control group (red signal) indicates baseline fluorescence, while time-resolved fluorescence profiles (dark green, blue, orange, and green) correspond to transformant populations harvested at 6, 8, 10, and 12 h post-inoculation, respectively.

To quantitatively assess promoter strength, we performed flow cytometry-based fluorescence profiling of transformant-grown 1 mM ATP supplementation. Relative fluorescence intensity normalized to wild-type controls revealed dynamic promoter activity across cultivation times (6-12 h). 6 h post-inoculation, the promoters were categorized into three tiers based on activity: SP2, SP4, SP6, SP19, SP20, SP34, SP36, and SP50 exhibited weak activity (FITC peak < 10^2^), SP10, SP30, and SP33 showed moderate strength (FITC peak ∼ 10^2^), and SP42, SP43, SP45, SP47, and SP49 demonstrated strong induction (FITC peak ∼ 10^3^). By 8 h, SP2, SP10, SP36, and SP47 retained moderate activity, whereas others declined to weak promoter levels. Prolonged cultivation to 10 h resulted in a further reduction in the activity, with only SP10 maintaining moderate expression. By 12 h, all promoters returned to low activity (Fig. 3 **and Figure S1**). Notably, SP10, SP36, and SP47 displayed sustained functionality throughout the time course (Fig. 3). Supplementation with 1 mM ATP markedly enhanced reporter expression fidelity, increasing both fluorescence intensity and the number of detectable transformants, indicative of improved thermotolerance and metabolic capacity. This systematic quantification establishes SP10, SP36, and SP47 as robust and time-stable promoters for engineering applications in *G. stearothermophilus* SL-1.

### 3.5 Heat resistance modification in G. stearothermophilus SL-1

**Fig. 4:**
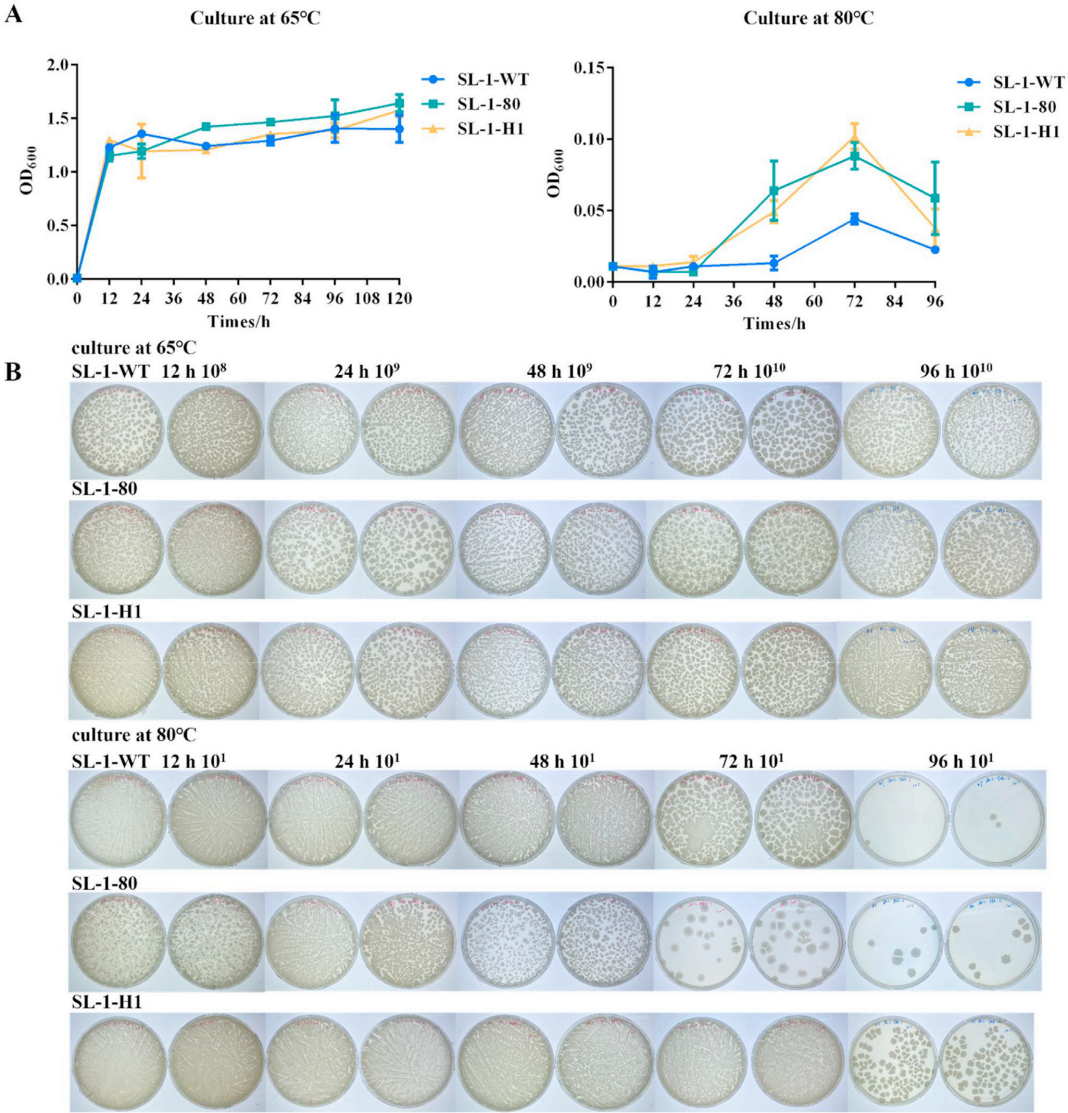
Comparative thermotolerance profiling of heat-adapted mutants (SL-1–80, SL-1-H1) versus wild-type SL-1 WT under multi-stress conditions revealed distinct phenotypic responses. (A) differential growth kinetics under sustained humid thermal stress is demonstrated: (A1) 65 °C/120 h cultivation and (A2) 80 °C/96 h exposure. (B) SL-1-H1 demonstrated superior viability and biomass retention compared to both SL-1-WT and SL-1–80, under both optimal (65 °C) and extreme (80 °C) temperature conditions.

We implemented a thermal adaptation laboratory evolution strategy to enhance the thermotolerance of the wild-type strain. Serial passaging in liquid medium with progressively elevated temperature (starting from 70 °C baseline) was alternated with solid-phase culture stabilization at each thermal threshold. After 20 iterative cycles, the evolved mutant *G. stearothermophilus* strain SL-1–80 exhibited an expanded growth celling of 80 °C, corresponding to a 10 °C improvement over the progenitor strain (Fig. 4). Nevertheless, the thermostability remained suboptimal for in situ microbial oil recovery applications, necessitating complementary genetic engineering approaches. Current understanding of thermophilic adaption mechanisms identifies heat shock proteins (HSPs) as critical mediators of high-temperature resilience. HSPs are structurally classified into five groups: HSP100 (> 100 kDa) [28], HSP90 (82-96 kDa) [29,30], HSP70 (67-76 kDa) [29], HSP60 (58-65 kDa) [31], and small HSPs (sHSPs, below 40 kDa) [32]. Functional studies highlight GroEL (HSP60 family) and DnaK (HSP70 family) as key molecular chaperones: DnaK ensures the fidelity of folding of nascent peptide chains, while GroEL repairs structurally compromised proteins under thermal denaturation [33].

Genomic annotation of *G. stearothermophilus* SL-1 identified 18 evolutionarily conserved thermotolerance determinants (**Table S7**), encompassing (i) molecular chaperones-small heat shock proteins(sHSPs: Hsp20 [34], Hsp33, and GroES), intermediate folding helpers (GrpE, GroEL, DnaK [35], DnaJ, HtpX [36]), and the Hsp100 family member ClpB, and (ii) cellular maintenance proteins (cysM [37] and murD [38]) critical for redox homeostasis and peptidoglycan biosynthesis under thermal stress. To synergistically amplify thermoresistance, we engineered a combinatorial overexpression library under strong constitutive promoters, leveraging the Red/ET recombineering scheme [[39], [40], [41], [42], [43]]. This plasmid library was electroporated into the SL-1–80 thermal-evolved strain (T_max_=80 °C), followed by stepwise thermal selection (1 °C increments) at 80 °C. After iterative screening, a singular hyperthermophilic mutant (SL-1-H1, T_max_=85 °C) emerged. Growth profiling revealed stark phenotypic divergence: while all strains exhibited comparable biomass yields at their shared optimum (65 °C), SL-1–80 and SL-1-H1 outperformed the wild type at 80 °C under moist heat (SL-1-H1 achieving 1.3-fold higher OD_600_ than SL-1–80; Fig. 4A). Under desiccated 80 °C stress, SL-1-H1 demonstrated superior viability and biomass retention (10-fold vs. SL-1–80; Fig. 4B).

**Fig. 5:**
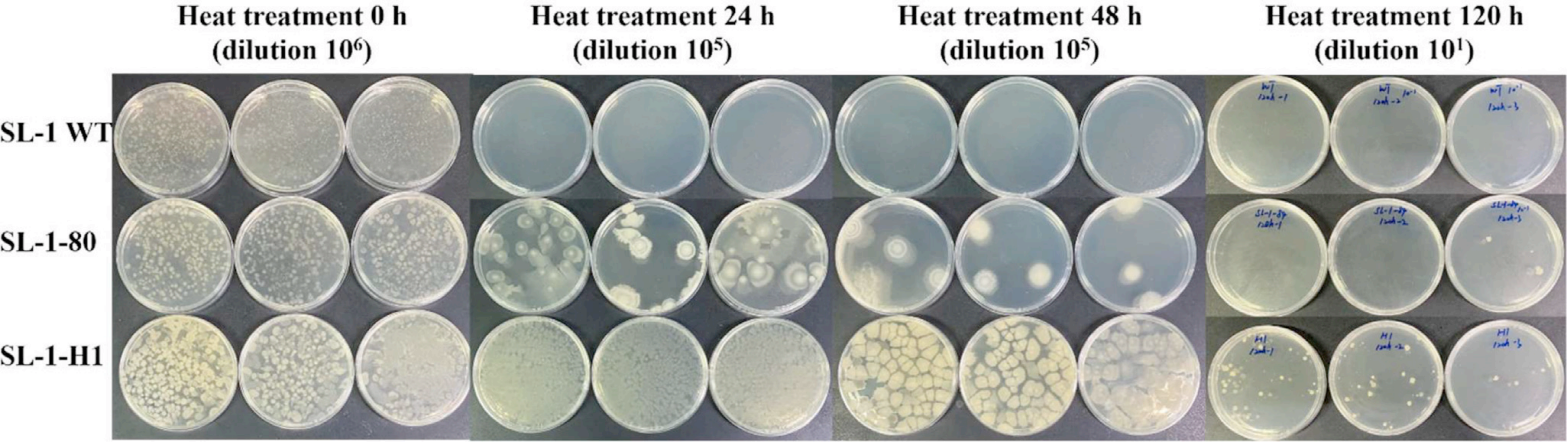
Thermotolerance assessment of SL-1–80, SL-1-H1, and SL-1 WT under dry heat stress revealed significant growth differentials. Illustration of the thermal challenge assays where normalized cultures (OD_600_=1.0, 65 °C) were subjected to extreme heat shock (85 °C), with viability monitored at 24 h intervals over 120 h. SL-1-H1 exhibits exceptional thermotolerance, maintaining viability under continuous 85 °C heat shock for 120 h.

Thermotolerance benchmarking at 85 °C revealed hierarchical survival dynamics among the strains. Pre-cultured at 65 °C to standardized OD_600_ (1.0), wild-type, SL-1–80, and SL-1-H1 exhibited differential persistence under lethal thermal stress (Fig. 5). Although no viable wild-type colonies survived beyond 24 h, SL-1–80 sustained culturability for 48 h, and SL-1-H1 maintained detectable CFUs through 120 h, demonstrating cumulative thermotolerance gains from adaptive evolution and combinatorial gene overexpression. To elucidate the genetic basis of SL-1-H1’s hyperthermophilia, we employed Gram-positive bacterial plasmid isolation followed by heterologous transformation into *E. coli* GB05, yielding 31 transformants. Restriction digested fingerprinting resolved seven distinct plasmid architectures, indicative of polyclonal plasmid coexistence in SL-1-H1 (**Figure S2**). NGS-based plasmidome profiling identified eight co-overexpressed loci: *murD, cysM, grpE, groES, hsp33, hslO, hrcA*, and *clpE.* Functional annotation confirmed the established roles of these genes in the heat-shock response pathway, with synergistic overexpression conferring the observed 5-fold extension in lethal temperature survival (85 °C stability). This modular gene stacking paradigm provides a translatable framework for enhancing industrial microbes under extreme bioprocessing conditions.

### 3.6 Comparative genomics analysis to identify heat-resistant mutation types

**Fig. 6:**
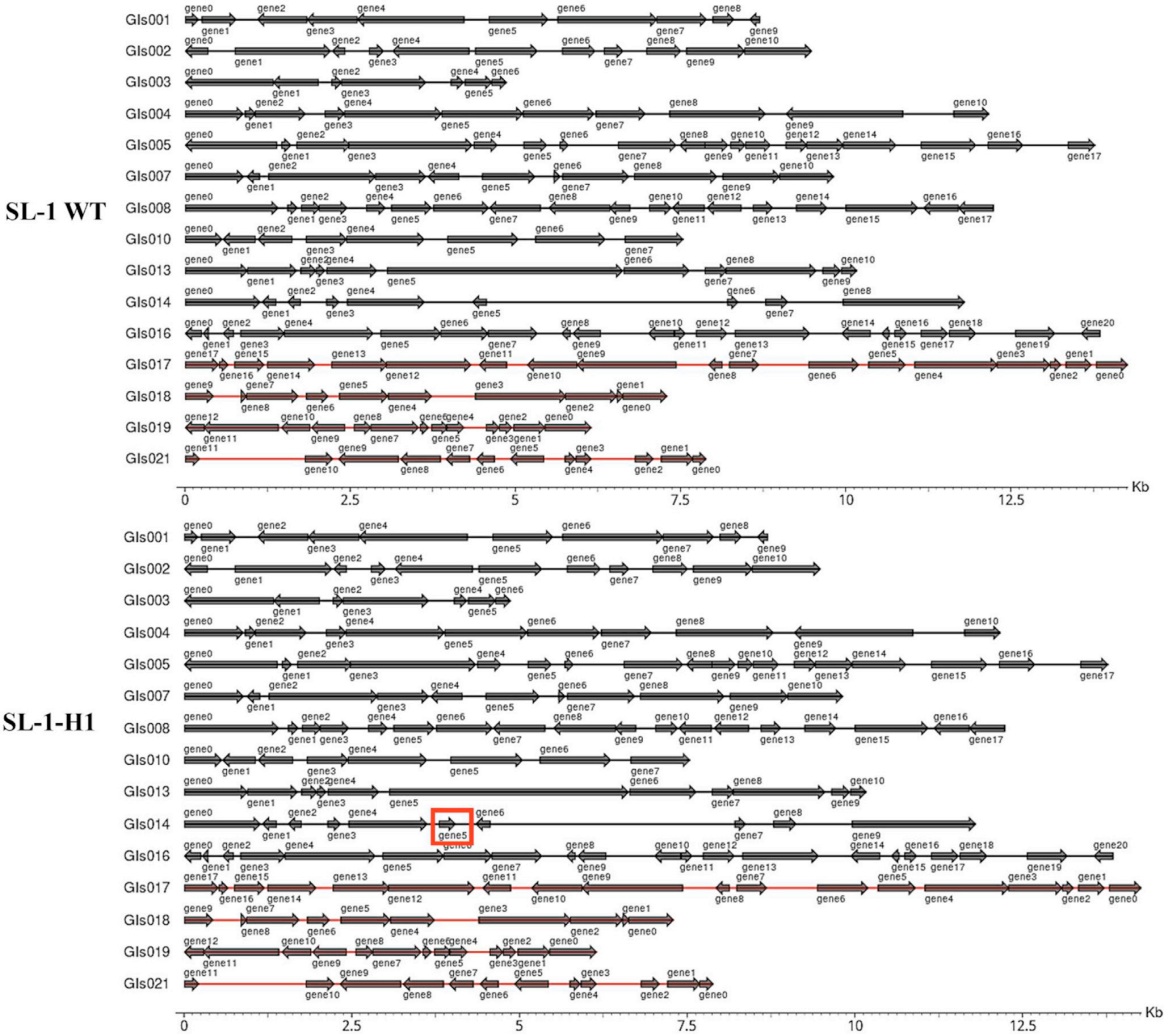
Genomic Variation Analysis of SL-1 WT and thermotolerant mutant SL-1-H1. Comparative genomic analysis revealed gene islands arrangements divergence between the wild-type strain SL-1 WT and its thermotolerant derivative SL-1-H1. A key distinction was identified within the highlighted genomic region (red box), where SL-1-H1 possesses an additional gene absent in the wild-type strain.

Whole-genome comparative analysis of *G. stearothermophilus* SL-1 WT and its thermotolerant derivative SL-1-H1 revealed targeted genomic restructuring (**Table S8**). Despite an overall genome size reduction (Δ513 bp), the mutant exhibited paradoxical increases including: (i) extended coding regions (+267 bp) with net gene gain (+2); (ii) compressed gene islands (−2516 bp) paradoxically containing additional ORF (+1). Detailed gene island profiling across 21 loci confirmed significant architectural divergence (Fig. 6), with one locus acquiring an extra gene. Transposon dynamics analysis identified three structural drivers: (i) translocation events (IS5377, *n* = 3; IS4, *n* = 3) with concomitant IS110 deletion (*n* = 1); (ii) one novel insertion each of IS5377 and IS4. These transposable elements, which are mediators of pathogenicity and environmental adaptation, likely originated through horizontal acquisition from bacteria, bacteriophages, or plasmids.

**Fig. 7:**
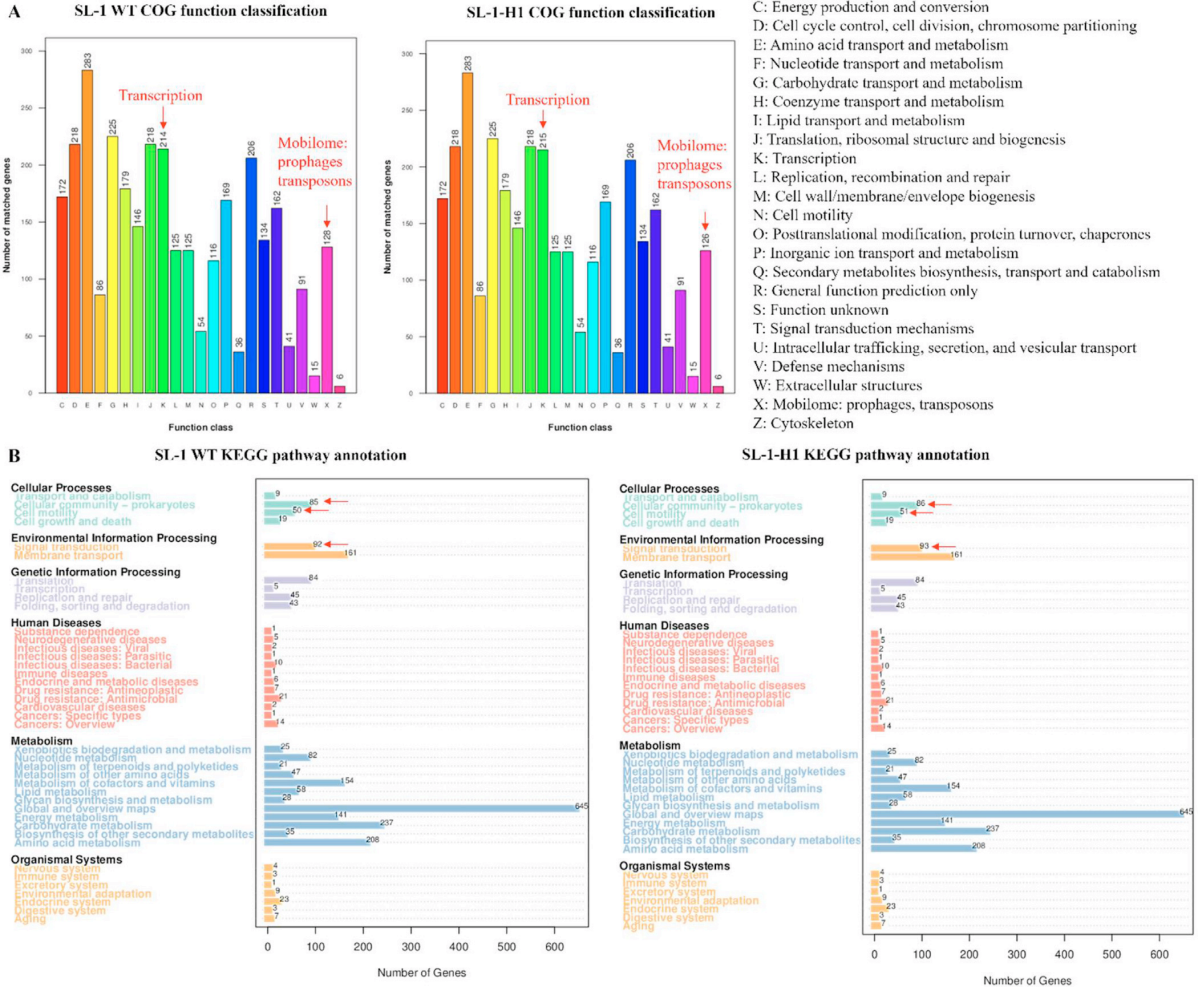
Comparative functional genomic analysis revealed divergence between the SL-1 WT and SL-1-H1 strains. COG classification alignment demonstrated that SL-1-H1 acquired one additional gene in category K (Transcription) while losing two genes in category X (Mobilome) compared to SL-1 WT (A). KEGG pathway analysis indicated modifications in SL1-H1, including altered gene counts involved in cellular community organization, motility apparatus, and signal transduction mechanisms relative to SL-1 WT (B).

Comparative effector-based profiling revealed conserved secretion systems and secondary metabolism clusters between strains. However, KEGG/COG-based annotation uncovered quantitative genomic shifts in SL-1-H1, including expanded cohorts of cellular community organizers (biofilm matrix/peptidoglycan-modifying enzymes), motility regulators (flagellar switch complexes), and transcriptional regulators (DNA-binding response factors) (Fig. 7). Mutational spectrum analysis delineated the following key modifications: (i) three revertant mutations restoring wild-type sequences in critical signaling nodes: *bglG* (transcription antiterminator), *fliY* (flagellar phosphatase), *sigD* (RNA polymerase sigma-D factor); (ii) one synonymous mutation (*hsdS*, EcoKI specificity subunit); and (iii) two frameshift disruptions: DUF47-family chaperone and HTH-domain transcriptional repressor.

Specifically, the BglG antiterminator revertant restored aromatic β-glucoside utilization under thermal stress, whereas the FliY phosphatase mutation enhanced chemotactic accuracy through phosphoregulation of flagellar receptors, particularly during early logarithmic and stationary phases. Concurrently, the sigma-D revertant enabled constitutive expression of flagellar biosynthesis genes throughout all growth phases, including structural components (*fliC, flgK*), motor complexes (*motA/B*), and chemosensory systems (*tar*), collectively enhancing swarming capacity and improving biofilm dynamics (**Figure S3**). Frameshift disruption of the DUF47-family chaperone was predicted to impair its regulatory function in stress adaptation and/or protein quality control pathways. The mutation in the HTH-domain transcriptional repressor relieved repression of genes associated with stress response, carbohydrate metabolism, and amino acid biosynthesis. These synergistic adaptations-encompassing metabolic plasticity (BglG and HTH-domain transcriptional repressor), motility optimization (FliY), and transcriptional reprogramming (Sigma-D, DUF47-family chaperone and HTH-domain transcriptional repressor)-demonstrate an evolutionarily coordinated strategy for thermal adaptation that facilitates both nutrient acquisition and motility-based environmental navigation under stress.

## 4 Discussion

The molecular mechanisms of thermoresistance in thermophiles provide critical insights for engineering microbial heat tolerance. In this study, we analyzed heat resistance genes in *G. stearothermophilus* SL-1 and constructed a combinatorial expression plasmid library containing cloned effector genes (*murD, cysM, grpE, groES, hsp33, hslO, hrcA, clpE*). Through adaptive laboratory evolution coupled with plasmid transformation, we obtained SL-1-H1, a mutant capable of sustained growth at 85 °C, an extension of 15 °C beyond the wild-type tolerance limit, representing one of the functional growth thresholds reported for engineered Gram-positive bacteria. This advance surpasses recent thermal engineering achievements in mesophiles (e.g., *E. coli* growth at 48.5 °C via ALE [26]). Our combinatorial overexpression strategy diverges from conventional single-gene approaches, revealing that synergistic activation of chaperone networks confers multi-layered protection [29,34]. Functional annotation revealed that these overexpressed genes encode molecular chaperones (GroES/ClpE), and effector proteins involved in cell wall synthesis (MurD), redox homeostasis (CysM/Hsp33), and transcriptional regulation (HrcA). Notably, their synergistic interactions with cellular components preclude the definitive attribution of thermotolerance to individual gene products. Future efforts should prioritize the elucidation of these cooperative networks to reveal the holistic mechanisms underlying bacterial thermoresistance.

Critical protocol optimization was required to establish the electrotransformation system for SL-1. Initial attempts using LB medium for both cell cultivation and post-electroporation recovery yielded no transformants, despite testing five plasmids, nine buffers, and four electric pulse conditions. System viability was achieved exclusively by replacing the recovery medium with 2× LBG (supplemented with 0.5 g/L glucose and 0.01 % BSA), while maintaining all previous operational conditions. This protocol shift highlights the need for nutrient-enhanced recovery environments to revive electrostressed cells. The dual supplementation operates synergistically: glucose provides immediately metabolizable carbon to fuel ATP-dependent membrane repair, while BSA likely stabilizes cytoplasmic proteins compromised by electroporative permeabilization. Our findings suggest that systematic integration of rapid-energy substrates and macromolecular stabilizers into transformation recovery media can be used to mitigate electroporation-induced metabolic burdens in bacterial systems. The establishment of high-efficiency electroporation (10^4^ CFU/µg DNA) resolves a longstanding limitation in *Geobacillus* genetic manipulation [16], outperforming existing protocols for thermophiles by two orders of magnitude [28].

However, the relative contributions of individual genes within our eight-gene cassette remain unclear, requiring gene-specific studies to deconvolute epistatic interactions. Plasmid stability in industrial environments (e.g., oil reservoirs) requires long-term validation, potentially necessitating genomic integration. While genome streamlining enhanced thermotolerance, transposon deletions risked purging beneficial MEOR traits, a risk partially mitigated by our approach. Future work should prioritize in situ validation, develop dynamically regulated heat-shock systems (e.g., thermosensitive promoters), and address ATP-dependency via engineered ATP-generation modules to boost thermostable biocatalysis.

In the mutant strain genome, all observed large-scale DNA fragment alterations were confined to transposons and their associated elements. The variation in the insertion and deletion frequencies between mutant and wild-type strain led to a genome size reduction in the mutant. We hypothesize that under combined thermal and environmental stress, bacteria preferentially eliminate nonfunctional exogenous sequences. This genome streamlining mechanism likely increases structural stability by removing mobile DNA components while alleviating metabolic costs during gene replication.

## 5 Conclusions

In summary, we engineered *G. stearothermophilus* SL-1-H1 as a robust thermophilic chassis through genomic stabilization and modular overexpression of thermoprotective genes, delivering the first efficient electrotransformation system for *Geobacillus* that enables ATP-enhanced heterologous expression at 85 °C. Furthermore, we identified eight synergistic thermotolerance genes whose combinatorial expression significantly extends the functional limits of bacterial heat resistance and obtained mechanistic insights into transposon-driven genome streamlining as an adaptive thermal stress response.

## Data Availability Statement

All relevant data supporting the findings of this study are available in this manuscript and the supplementary materials.

## CRediT authorship contribution statement

**Jie Cui:** Writing – original draft, Data curation, Conceptualization. **Caifeng Li:** Data curation. **Gongze Cao:** Methodology. **Yuxia Wu:** Resources. **Shouying Xu:** Project administration. **Youming Zhang:** Writing – review & editing, Supervision. **Xiaoying Bian:** Writing – review & editing, Supervision. **Qiang Tu:** Writing – review & editing, Supervision. **Wentao Zheng:** Writing – review & editing, Supervision, Project administration.

## Declaration of Competing Interest

The authors declare the following financial interests/personal relationships which may be considered as potential competing interests: Given his role as Editor-in-Chief, Dr. Youming Zhang, had no involvement in the peer-review of this article and has no access to information regarding its peer-review. Full responsibility for the editorial process for this article was delegated to Dr. Shengbiao Hu.

## Appendix Supplementary materials

Image, application 1
