# Supplementary material for: Screening of molecular elements and improvement of heat resistance in a thermophilic bacterium
Source: Eng Microbiol. 2025 Jul 22;5(3):100225. doi: 10.1016/j.engmic.2025.100225 (PMC12967825; doi:10.1016/j.engmic.2025.100225)
Supplement: Supplementary file 1 [file mmc1.docx]

**Supplementary Information for**

**Screening of Molecular Elements and Improvement of Heat Resistance in a Thermophilic Bacterium**

**Jie Cui^1, 2^, Caifeng Li^2^, Gongze Cao^2^, Yuxia Wu^1^, Shouying Xu^1^, Youming Zhang^1, 3^, Xiaoying Bian^1, *^, Qiang Tu^1, 3, *^, Wentao Zheng^1, 4, *^**

^1^ State Key Laboratory of Microbial Technology, Shandong University, No. 72 Binhai Road, Qingdao, 266237, China

^2^ Microbial Oil Recovery Research Institute, Research Institute of Petroleum Engineering, Shengli Oilfield Company, Sinopec, Dongying, 257000, China

^3^ Institute of Synthetic Biology Industry, Hunan University of Arts and Science, Changde 415000, China

^4^ Shenzhen Research Institute of Shandong University, A301 Virtual University Park in South District of Shenzhen, Guangdong, 518000, P. R. China

* To whom correspondence should be addressed. Email: zwt12138@foxmail.com

Correspondence may also be addressed to Qiang Tu, Email: tuqiang1986@163.com; Xiaoying Bian, Email: bianxiaoying@sdu.edu.cn


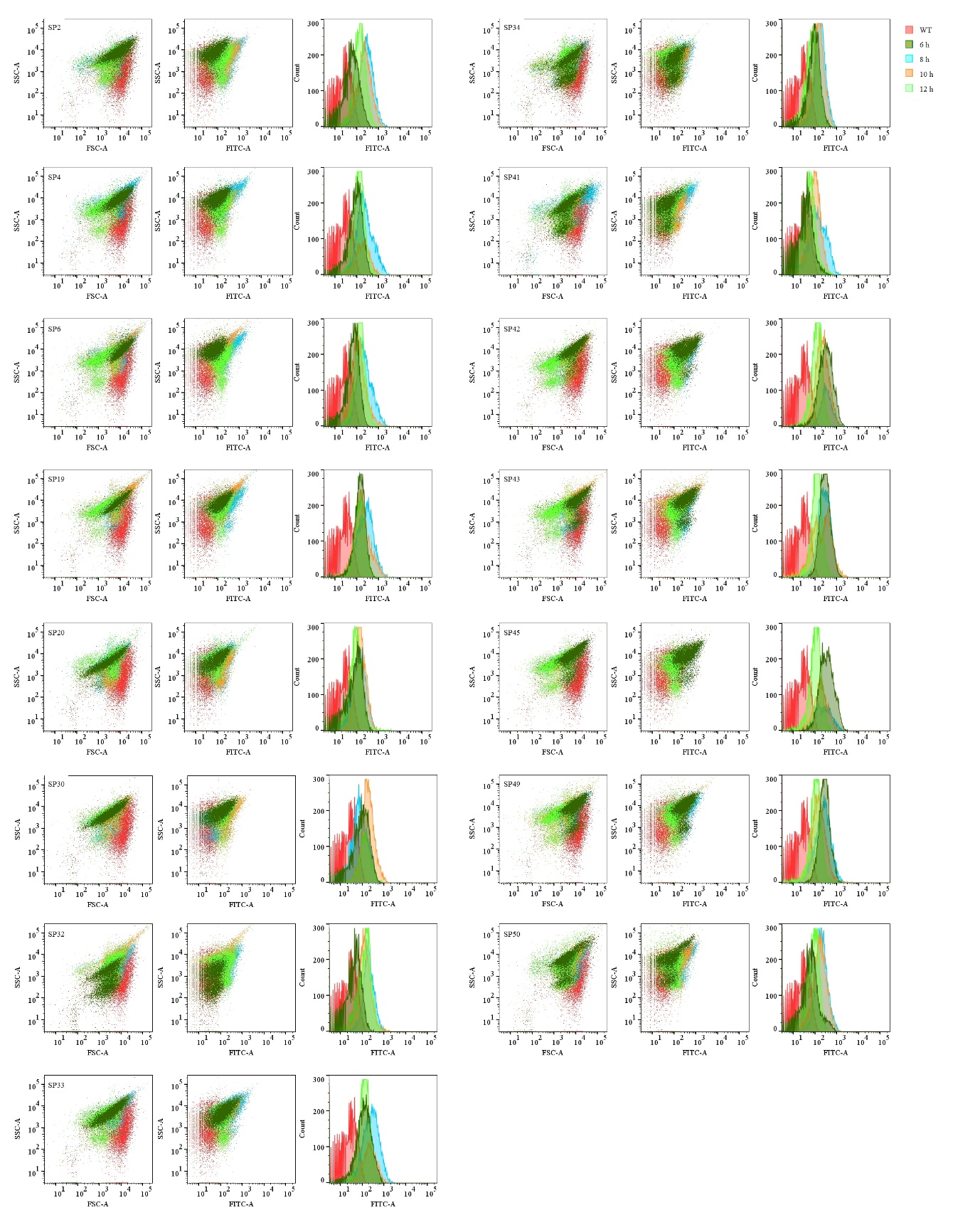


**Figure S1**. Quantification of promoter activity in *Geobacillus stearothermophilus* SL-1 WT under 1 mM ATP induction at distinct growth phases was performed via flow cytometric analysis. Among 18 screened promoters, SP2, SP4, SP6, SP19, SP20, SP30, SP32-SP34, SP41-SP43, SP45, SP49, SP50 constructs were presented. The control group (red signal) represents baseline fluorescence, while time-resolved fluorescence profiles (dark green, blue, orange, and green) correspond to transformant populations harvested at 6, 8, 10, and 12 hours post-inoculation, respectively.


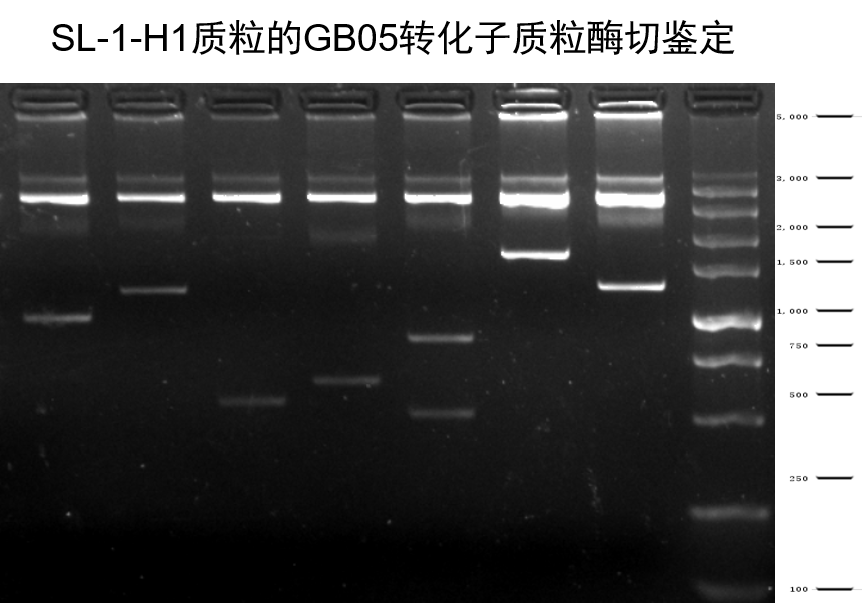


**Figure S2**. Restriction enzyme digestion profile of plasmids carried in E. coli GB05, derived from the SL-1-H1 mutant strain


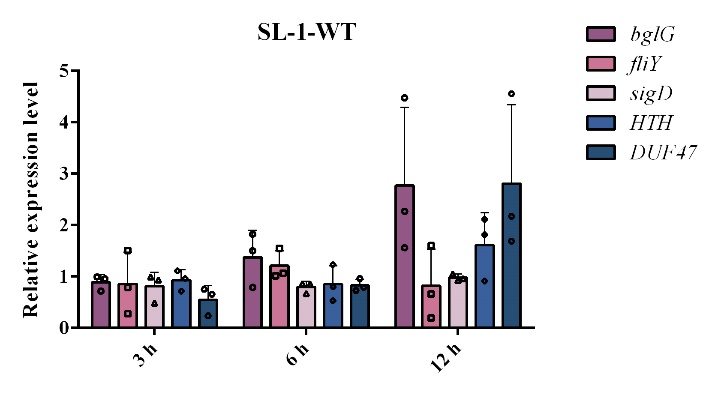

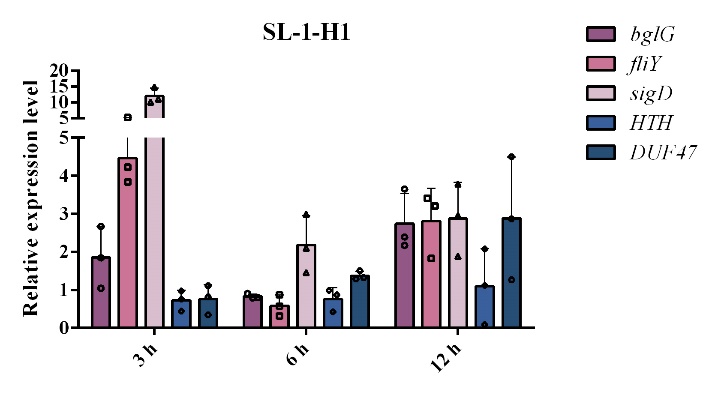


**Figure S3**. Transcriptional levels of target genes were compared between the wild-type strain SL-1-WT and its thermotolerant mutant SL-1-H1 at three growth stages: early-log phase (3 h), mid-log phase (6 h), and stationary phase (12 h). The NADPH dehydrogenase gene was used as an internal control for normalization.

**Table S1**. Summary of culture medium used in this study

| Name | Content |
| --- | --- |
| Low-salt LB medium | peptone 10 g/L, yeast extract 5 g/L, NaCl 1 g/L |
| 2 × LBG medium | yeast extract 10 g/L, peptone 20 g/L, glucose 5 g/L, add deionized water to 1 L, 115°C, 30 min, high pressure steam sterilization. Deionized water is used to prepare 10 % (w /v) BSA solution, filter sterilize, and add to the cooled sterilized material to a final concentration of 0.01 %. |

Solid culture medium is prepared by adding 1.2 % agar powder to the corresponding liquid culture medium

**Table S2**. Antibiotic susceptibility of *G. stearothermophilus* SL-1

| Antibiotic Name | Minimum inhibitory concentration/μg·mL^-1^ | Category |
| --- | --- | --- |
| Chloramphenicol (Cm) | 15 | Chloramphenicol antibiotics |
| Tetracycline (Tet) | 10 | Tetracycline antibiotics |
| Erythromycin (Em) | 10 | Macrolide antibiotics |
| Apramycin (Apra) | 10 | Aminoglycoside antibiotics |
| Kanamycin (Km) | 80 |  |
| Gentamicin (Genta) | 10 |  |
| Streptomycin (Sm) | 15 |  |

**Table S3**. Parameters for establishing electroporation *of G. stearothermophilus* SL -1

| Electroporation  Bacterial growth status | Mid-exponential growth | | | | Late exponential growth | | | | | Stable period | | | |
| --- | --- | --- | --- | --- | --- | --- | --- | --- | --- | --- | --- | --- | --- |
| System temperature during electroporation | 25 ℃ （Room Temperature） | | | | | | | 4℃ | | | | | |
| Buffer | water | 10% glycerol | 20% glycerol | | | 20% sucrose | Water + 2 mM HEPES | | 10% glycerol + 2mM HEPES | | 20% glycerol + 2mM HEPES | | 20% sucrose + 2mM HEPES |
| Electroporation voltage | 2500 V  (2 mm electric shock cup) | | | 2000 V  (2 mm electric shock cup) | | | | 1350 V  (1 mm electric shock cup) | | | | 1250 V  (1 mm electric shock cup) | |
| Recovery medium | LB | | | | | | | 2 × LBG | | | | | |
| Recovery temperature | 6 0℃ | | | | 55℃ | | | | | 48℃ | | | |
| Recovery time | 1 2 h | | | | 2 h | | | | | 1 h | | | |

**Table S4**. Candidate promoters identified from highly expressed genes in *G. stearothermophilus* SL-1

| **promoter** | **GenenID** | **Annotation** | **length** |
| --- | --- | --- | --- |
| SP1 | K4GL003514 | S-layer homology domain | 302 |
| SP2 | K4GL002510 | Stage IV sporulation protein A (spore_IV_A) | 112 |
| SP3 | K4GL002110 | Aldehyde dehydrogenase family | 70 |
| SP4 | K4GL003708 | Peptidase family M23 | 103 |
| SP5 | K4GL000655 | PrkA serine protein kinase C-terminal domain | 57 |
| SP6 | K4GL000115 | Elongation factor Tu GTP binding domain | 122 |
| SP7 | K4GL000968 | FAD dependent oxidoreductase | 76 |
| SP8 | K4GL002111 | pfkB family carbohydrate kinase | 37 |
| SP9 | K4GL002878 | Thioredoxin domain or Pyridine nucleotide-disulphide oxidoreductase | 24 |
| SP10 | K4GL000204 | Aldehyde dehydrogenase family | 100 |
| SP11 | K4GL001875 | hypothetical protein CDS | 443 |
| SP12 | K4GL000924 | hypothetical protein CDS | 150 |
| SP13 | K4GL001873 | hypothetical protein CDS | 198 |
| SP14 | K4GL002974 | Bacterial regulatory proteins, luxR family | 259 |
| SP15 | K4GL003710 | EPSP synthase (3-phosphoshikimate 1-carboxyvinyl-transferase) | 31 |
| SP16 | K4GL000225 | Subtilase family | 124 |
| SP17 | K4GL000948 | hypothetical protein CDS | 302 |
| SP18 | K4GL001741 | Protein of unknown function (Tiny_TM_bacill) | 157 |
| SP19 | K4GL000167 | SIS domain\|Glutamine amidotransferase domain | 525 |
| SP20 | K4GL000947 | hypothetical protein CDS | 16 |
| SP21 | K4GL003815 | Alanine dehydrogenase/PNT, C or N-terminal domain | 117 |
| SP22 | K4GL003760 | ArgK protein\|Methylmalonyl-CoA mutase\|B12 binding domain | 80 |
| SP23 | K4GL000266 | TCP-1/cpn60 chaperonin family | 102 |
| SP24 | K4GL003542 | Aldehyde dehydrogenase family | 198 |
| SP25 | K4GL002052 | hypothetical protein CDS | 513 |
| SP26 | K4GL003146 | DeoR-like helix-turn-helix domain | 170 |
| SP27 | K4GL001684 | Cytochrome C and Quinol oxidase polypeptide I | 21 |
| SP28 | K4GL001467 | Aconitase C-terminal domain\|Aconitase family (aconitate hydratase) | 138 |
| SP29 | K4GL001414 | Outer spore coat protein E (CotE) | 27 |
| SP30 | K4GL000088 | UvrB/uvrC motif\|C-terminal, D2-small domain, of ClpB protein\|AAA domain (Cdc48 subfamily) \|ATPase family associated with various cellular activities (AAA)\|Clp amino terminal domain, pathogenicity island component | 50 |
| SP31 | K4GL003166 | Phosphoenolpyruvate carboxykinase | 358 |
| SP32 | K4GL001756 | AMP-binding enzyme C-terminal domain\|AMP-binding enzyme | 74 |
| SP33 | K4GL002879 | AhpC/TSA family | 357 |
| SP34 | K4GL003436 | Sigma 54 modulation\|S30EA ribosomal protein C terminus | 277 |
| SP35 | K4GL000110 | RNA polymerase Rpb1, domain 3 | 138 |
| SP36 | K4GL001165 | Polypeptide deformylase | 485 |
| SP37 | K4GL003044 | Citrate synthase | 266 |
| SP38 | K4GL003043 | Isocitrate/isopropylmalate dehydrogenase | 50 |
| SP39 | K4GL002665 | 2-oxoacid dehydrogenases acyltransferase (catalytic domain)\|e3 binding domain\|Biotin-requiring enzyme | 198 |
| SP40 | K4GL000336 | HNH endonuclease | 369 |
| SP41 | K4GL002767 | Iron/manganese superoxide dismutases | 157 |
| SP42 | K4GL002976 | FAD binding domain\|Fumarate reductase flavoprotein C-term | 29 |
| SP43 | K4GL000114 | Elongation factor Tu domain | 235 |
| SP44 | K4GL003727 | ATP synthase alpha/beta family | 27 |
| SP45 | K4GL001762 | Carboxyl transferase domain | 110 |
| SP46 | K4GL000109 | RNA polymerase Rpb2 | 32 |
| SP47 | K4GL002902 | LysM domain | 158 |
| SP48 | K4GL003199 | Alpha amylase, catalytic domain\|Carbohydrate-binding module 48 (Isoamylase N-terminal domain) | 198 |
| SP49 | K4GL000757 | Isocitrate lyase family | 168 |
| SP50 | K4GL000882 | Bacterial extracellular solute-binding proteins, family 5 Middle | 404 |

**Table S5**. Fluorescence detection in transformants at different culture times

| Cultivation time | Transformant types that produces fluorescence |
| --- | --- |
| 6 h |  |
| 8 h | P2, P4, P49 |
| 10h | P2, P10, P34, P43 |
| 12h | P2 |

**Table S6**. Fluorescence detection in transformants under different ATP concentrations and cultivated time

| ATP concentrations | Cultivation time | Transformant types that produce fluorescence |
| --- | --- | --- |
| 0 mM | 6 h |  |
| 0 mM | 8 h | SP2、SP4、SP49 |
| 0 mM | 10 h | SP2、SP10、SP34、SP43 |
| 0 mM | 12 h | SP2 |
| 0.2 mM | 6 h | SP30 |
| 0.2 mM | 8 h | SP2、SP4、SP49 |
| 0.2 mM | 10 h | SP2、SP10、SP34、SP43 |
| 0.2 mM | 12 h | SP2 |
| 0.4 mM | 6 h | SP2、SP47 |
| 0.4 mM | 8 h | SP2、SP4、SP36、SP47、SP49 |
| 0.4 mM | 10 h | SP2、SP10、SP34、SP43 |
| 0.4 mM | 12 h |  |
| 0.6 mM | 6 h | SP2、SP10 |
| 0.6 mM | 8 h | SP2、SP4、SP10、SP49 |
| 0.6 mM | 10 h | SP2、SP10、SP34、SP36、SP42、SP43 |
| 0.6 mM | 12 h |  |
| 0.8 mM | 6 h | SP2、SP10、SP47 |
| 0.8 mM | 8 h | SP2、SP4、SP10 SP49 |
| 0.8 mM | 10 h | SP2、SP10、SP42、SP43 |
| 0.8 mM | 12 h |  |
| 1 mM | 6 h | SP2、SP10、SP30、SP45、SP47 |
| 1 mM | 8 h | SP2、SP10、SP20、SP33、SP36、SP49 |
| 1 mM | 10 h | SP10、SP34、SP36、SP42 |
| 1 mM | 12 h | SP4、SP6、SP10、SP19、SP20、SP32、SP33、SP34、SP41、SP45、SP49 |
| 1.2 mM | 6 h |  |
| 1.2 mM | 8 h | SP10 |
| 1.2 mM | 10 h | SP10、SP34、SP43 |
| 1.2 mM | 12 h |  |

**Table S7**. Temperature stress-related proteins in the SL-1 genome of *G. stearothermophilus*

| Serial number | Temperature stress-related proteins | Gene size/bp |
| --- | --- | --- |
| 1 | Molecular chaperone protein DnaJ | 1146 |
| 2 | Molecular chaperone protein DnaK | 1818 |
| 3 | Molecular chaperone protein Hs1O | 903 |
| 4 | Molecular chaperone protein GroES | 285 |
| 5 | Molecular chaperone protein GroEL | 1623 |
| 6 | Molecular chaperone protein GrpE | 672 |
| 7 | Heat shock protein Hsp20 | 540 |
| 8 | Heat shock protein Hsp33 | 318 |
| 9 | Heat shock protein HtpX | 1875 |
| 10 | Heat-induced transcriptional repressor HrcA | 1035 |
| 11 | ATP-dependent Clp protease ATP-binding subunit | 612 |
| 12 | ATP-dependent Clp protease ATP-binding subunit ClpX | 1266 |
| 13 | Molecular chaperone protein ClpB | 2586 |
| 14 | ATP-dependent Clp protease ATP-binding subunit ClpE | 2136 |
| 15 | Cysteine synthase CysM | 927 |
| 16 | Glutamate ligase murD | 1356 |
| 17 | Heme chaperone protein HemW | 1254 |
| 18 | Cold shock protein CspB | 201 |

**Table S8**. Comparison of parameters at the genome level between wild type strain SL-1 WT and thermotolerant mutant SL-1-H1

| Sample name | Genome size (bp) | Number of genes | Coding region length (bp) | Average gene length (bp) | Genomics Islands total length (bp) |
| --- | --- | --- | --- | --- | --- |
| SL-1 WT | 3,611,261 | 3,850 | 3,107,286 | 807 | 351,054 |
| SL-1-H1 | 3,610,748 | 3,852 | 3,107,553 | 807 | 348,538 |

**Table S9**. Summary of antibiotics and inducers used in *E. coli* in this study

| Antibiotics/inducers | Storage concentration  (mg·mL^-1^) | Working concentration (μg·mL^-1^) | |
| --- | --- | --- | --- |
|  |  | Liquid culture medium | Solid culture medium |
| Arabinose (Ara) | 100 | 1400 | / |
| Apramycin (Apra) | 50 | 10 | 15 |
| Chloramphenicol (Cm) | 50 | 10 | 15 |
| Gentamicin (Gentamicin) | 50 | 4 | 8 |
| Kanamycin (Km) | 50 | 10 | 20 |

Reference

1. Wu LJ, Welker NE. Protoplast transformation of *Bacillus stearothermophilus* NUB36 by plasmid DNA. J Gen Microbiol, 1989, 135(5): 1315-1324.
